# Polymethacrylate Coated Electrospun PHB Fibers as a Functionalized Platform for Bio-Diagnostics: Confirmation Analysis on the Presence of Immobilized IgG Antibodies against Dengue Virus

**DOI:** 10.3390/s17102292

**Published:** 2017-10-09

**Authors:** Samira Hosseini, Pedram Azari, Martín F. Jiménez-Moreno, Aida Rodriguez-Garcia, Belinda Pingguan-Murphy, Marc J. Madou, Sergio O. Martínez-Chapa

**Affiliations:** 1 Escuela de Ingeniería y Ciencias, Tecnologico de Monterrey, Ave. Eugenio Garza Sada 2501, Monterrey 64849, NL, Mexico; samira.hosseini@itesm.mx (S.H.); A00904856@itesm.mx (M.F.J.-M.); 2 Department of Biomedical Engineering, Faculty of Engineering, University of Malaya, Kuala Lumpur 50603, Malaysia; pedram.azari@gmail.com; 3 Centre for Applied Biomechanics, Faculty of Engineering, University of Malaya, Kuala Lumpur 50603, Malaysia; 4 Instituto de Biotecnologia, Facultad de Ciencias Biologicas, Universidad Autonoma de Nuevo Leon, San Nicolas de los Garza 66455, NL, Mexico; aida.rodriguezgrc@uanl.edu.mx; 5 Department of Biomedical Engineering, University of California, Irvine, CA 92697, USA; mmadou@uci.edu; 6 Department of Mechanical and Aerospace Engineering, University of California, Irvine, CA 92697, USA

**Keywords:** protein immobilization confirmation analyses, electrospun fiber mats, surface roughness, FTIR finger print region, functional groups

## Abstract

In this article, a combination of far field electrospinning (FFES) and free-radical polymerization has been used to create a unique platform for protein immobilization via the physical attachment of biomolecules to the surface of the fiber mats. The large specific surface area of the fibers with its tailored chemistry provides a desirable platform for effective analyte-surface interaction. The detailed analysis of protein immobilization on a newly developed bio-receptive surface plays a vital role to gauge its advantages in bio-diagnostic applications. We relied on scanning electron microscopy (SEM), diameter range analysis, and X-ray photoelectron spectroscopy (XPS), along with thermal gravimetric analysis (TGA), water-in-air contact angle analysis (WCA), Fourier transform infrared spectroscopy (FTIR), and atomic force microscopy (AFM) to study our developed platforms and to provide valuable information regarding the presence of biomolecular entities on the surface. Detailed analyses of the fiber mats before and after antibody immobilization have shown obvious changes on the surface of the bioreceptive surface including: (i) an additional peak corresponding to the presence of an antibody in TGA analysis; (ii) extra FTIR peaks corresponding to the presence of antibodies on the coated fiber platforms; and (iii) a clear alteration in surface roughness recorded by AFM analysis. Confirmation analyses on protein immobilization are of great importance as they underlay substantial grounds for various biosensing applications.

## 1. Introduction

The development of a unique surface for improved protein immobilization is critical as, unlike DNAs, proteins are heterogeneous, unstable, and have a three-dimensional (3D) structure that cannot be amplified for detection [1,2]. From that perspective, the fabrication and processing of sensitive bio-receptive platforms with specific physical and chemical properties for enhanced protein immobilization has drawn a great deal of interest in the bio-sensing domain [3,4]. While the readout of the detection signal intensity itself provides indirect information about the quality of the protein immobilization on the surface, the application of different analytical techniques can deliver direct evidence on the successful protein attachment. A large number of different techniques have been employed to provide confirmation for the immobilization of a wide variety of proteins on bio-sensing platforms [5,6,7,8]. In particular, the analysis of a layer of antibodies immobilized on a surface has attracted special attention since it acts as the foundation for the immobilizations of the biomolecular chain that can subsequently lead to the bio-recognition of the target analyte [5,6,7,8].

Since the advent of the electrospinning technique in 1887, numerous types of fibers have been developed for a variety of applications such as regenerative medicine, controlled drug release, molecular separation, wound healing applications, and biomaterials engineering [1,9,10,11,12,13]. Electrospun fiber mats have also proven to be suitable candidates for biosensors due to their high specific surface area, controllable porosity, interconnectivity, and low cost [14,15]. Electrospun fibers of different classes can be integrated into the microfluidic platforms such as lab-on-a-chip (LOC) and/or lab-on-a-compact disk (LOCD) devices for extreme point of care (EPOC) [16]. Although the physical properties of such versatile fiber mats promote biomolecular interaction, the presence of active functional groups such as carboxyl (–COOH), amine (–NH_2_), hydroxyl (–OH), and/or sulfhydryl (–SH) in the structure of the fibers can further facilitate analyte-surface interaction in an efficient manner [8,14,17]. These functional groups involve one or more of the major molecular forces such as ionic attraction, van der Waals’ forces, hydrophobic interaction, and hydrogen bounding (H-bonding) in interacting with the biomolecular entities of interest [3,18,19].

In this work, a new class of fiber-based material was fabricated by combining far field electrospinning (FFES) with free-radical polymerization. Electrospun polyhydroxybutyrate (PHB) fibers were dip-coated in polymethyl methacrylate-co-methacrylic acid abbreviated as poly(MMA-co-MAA) in order to introduce active –COOH functional groups to the structure of the fiber material [14]. This study is dedicated to the analyses of the electrospun PHB fibers in the pure form (uncoated) and co-polymer coated before and after protein immobilization and the effect of the coating on the performance of the bio-receptive surfaces. The synergy of the mentioned techniques resulted in a unique platform that combines the vast specific surface area of the fiber mat structure and the presence of –COOH functional groups derived from co-polymer coated segments. The fiber-based platforms were tested for the bio-recognition of dengue virus (DENV) as the target analyte via a sandwich enzyme-linked immunosorbent assay (ELISA). Details of the immunoassay and detection performance of the fibers were previously reported [14]. As the main focus of this study, different analytical techniques were employed, namely scanning electron microscopy (SEM), diameter range analysis, X-ray photoelectron spectroscopy (XPS), thermal gravimetric analysis (TGA), water-in-air contact angle analysis (WCA), Fourier transform infrared spectroscopy (FTIR), and atomic force microscopy (AFM), in order to study the effect of a coating on protein immobilization from a more fundamental point of view. Those techniques were used to initially confirm the successful dip-coating of the fiber mats by the specifically synthesized co-polymer composition and to subsequently study traces of the immobilized proteins within the network of the coated electrospun fibers. We found clear evidence for the effective coating and the presence of immobilized antibodies on the surface of the fibers. Detailed analyses of the newly developed bio-receptive platforms before and after immobilization lead to a better understanding of the designed surfaces and further validate their performance in bio-diagnostic applications.

## 2. Materials and Method

### 2.1. Chemicals and Reagents

Methyl methacrylate (MMA), methacrylic acid (MAA), and PHB were purchased from Sigma (St. Louis, MO, USA). MMA monomer was purified by distillation prior to application in the free-radical polymerization reaction. Other supplies were used as received. Tetrahydrofuran (THF) was used as a solvent in the polymerization reaction and coating procedure, and dimethylformamide (DMF) was purchased from Merck (Darmstadt, Germany). Chloroform (Chl) and azobisisobutyronitrile (AIBN), the initiator in the polymerization reaction, were purchased from Friedemann Schmidt Chemical (Berlin, Germany).

### 2.2. Fabrication of Uncoated and Coated Electrospun PHB Fibers

PHB fibers were fabricated using the far field electrospinning (FFES) technique previously described [14,20,21]. In brief, PHB solution (10% *w*/*v* in Chl/DMF with a ratio of 9:1) was ejected from a blunt needle depositing the electrospun fibers on a target aluminum plate (collector) using a voltage of 12 kV. The distance between the needle and the collector was 18 cm. The electrospun fiber mat with an approximate thickness of ~500 µm was peeled off from the collector and coated with poly(MMA-co-MAA) [14]. Poly(MMA-co-MAA) was synthesized in a free-radical polymerization reaction by using THF as a solvent and AIBN as an initiator. The molar ratio of the monomer was chosen to be 9:1 for MMA and MAA [14]. A straightforward method of dip-coating (immersing PHB fiber mats into the 5% polymer solution in THF for 3 s) was used for the polymer coating of the electrospun fibers. Co-polymer coated electrospun PHB fiber mats were then cut into circular shapes of a 6 mm diameter and placed inside a 96-well plate to be used for the detection of DENV via a sandwich assay. The methodology for conducting the sandwich ELISA, the chemicals and reagents used, as well as the biomolecular sequence, can be found in great detail in our previous publication [3,14]. 

### 2.3. Application of the Fibers in Sandwich Immunoassay

Fiber mats (coated and uncoated) were cut into circular shapes with a diameter of 6 mm and placed into a conventional 96-well plate for the detection of DENV. Capture antibodies (Goat IgG anti DENV, SC-325014, Santa Cruz, CA, USA) were immobilized (concentration: 3.5 × 10^3^ p.f.u/mL) on the surface of the chemically designed fibers via direct immobilization techniques relying on the physical forces that connect antibodies to the surface functional groups. After capture antibodies were immobilized, the surface was blocked to avoid non-specific binding. The blocking step was followed by the attachment of DENV, primary, and eventually secondary labeled antibodies. The detection signal was recorded through the colorimetric readout of the samples. Details of the detection and immobilization procedures can be found in our previous publications [14]. 

### 2.4. Analysis of the Electrospun PHB Fibers before and after Co-Polymer Coating and Protein Immobilization

The morphology of the coated and uncoated fiber mats was analyzed by SEM equipped with a field emission gun (JEOL, JSM7600F, Peabody, MA, USA) operated at an accelerating voltage of 0.5 kV. Fiber samples were gold-coated to avoid surface charging. Frontal and cross-section views were recorded in secondary electron mode. Pore size measurements were performed on 100 ± 2 random pores from five different samples to obtain the average pore size of uncoated and coated PHB fibers. 

Diameter range analysis was performed based on 100 readings for each sample from three different SEM images performed by ImageJ software. The beads, fiber overlapping, and crossings were avoided in the measurements. Only the fibers with clear borders were measured to reduce the chance of any errors.

The XPS analysis was performed by using a Quantera SXMtm instrument from Ulvac-PHI. The measurements were recorded by using monochromatic AlKα-radiation with an analysis depth of approximately 7 nm. The take-off angle was 45° for the wide-scan measurements and the size of the analyzed spots was 300 μm × 500 μm. Narrow-scans were used for the precise identification of chemical states following the standard conversion of the peak positions to the corresponding atomic concentrations. 

Thermal analysis was performed by TGA on uncoated and polymer coated PHB fibers to identify the weight loss (W, %) of the compounds and to determine the temperature for the onset of degradation (T_OD_). The apparatus used in these experiments was a SETARAM SETSYS evolution thermo-gravimeter (model 1200, Lyon, France). Samples were examined under an inert atmosphere of helium (5.5 purity, SIAD TP) while the gas flow was kept constant at 30 cm^3^/min at a pressure of 101.325 kPa in all experiments. A platinum crucible with an approximate weight of 4 mg was used as a sample holder. The temperature in the TGA experiments was increased at a rate of 20 °C/min starting from ambient temperature and increasing up to 900 °C. Polymer coated PHB fibers were also examined after the immobilization of antibodies against DENV to investigate traces of the biomolecular entities on the surface. Coated samples used for protein immobilization were dried prior to TGA analysis. Coated fibers that were not used in the protein immobilization process were also incubated in coating buffer (the same buffer that was used in the actual assay for dilution of the capture antibodies) for 2 h and dried in the oven of 60 °C prior to TGA analysis in order to be used as controls. 

Water-in-air contact angle analysis was performed with droplets of distilled water (0.1 µL) deposited on the coated and uncoated surfaces of fiber mats and measurements were made 30 s after deposition. The WCA experiment was carried out using the sessile drop method by a Dataphysics contact angle system (OCA) at ambient temperature. Contact angles were measured for the droplets deposited on the center and four corners of the sample and 15 readouts were recorded (three samples for each composition). The standard deviations were found to be negligible. 

Both coated and uncoated electrospun fibers were analyzed by FTIR to characterize the chemical structure of the polymers. Infrared spectra were recorded in attenuated total reflectance mode by ATR-FTIR using a Perkin Elmer (model 400, Waltham, MA, USA) in the wavelength range of 400–4000 cm^−1^. In order to have a clear comparison between the spectra before and after protein immobilization, coated PHB fibers were also analyzed by FTIR once capture antibodies against DENV were immobilized on the surfaces. Samples were fully dried prior to FTIR analysis. Nevertheless, the same incubation in coating buffer (2 h) and subsequent drying process (in the oven of 60 °C) were also performed on coated PHB fibers that had not been used for protein immobilization. 

The surface topology of the uncoated and polymer coated PHB fibers was recorded by AFM in a non-contact mode. The apparatus used in these experiments (Ambios, Q scope, Totnes, UK) established the mean roughness (Ra), root mean-square roughness (Rq), and total roughness (Rt) for all the analyzed samples. Coated electrospun fibers after antibody immobilization were also analyzed by AFM to identify changes in the roughness parameters caused by the presence of proteins at the uppermost surface of the coated fibers. 

## 3. Results and Discussion 

In order to obtain a better understanding of the interface between the biomolecular entities and the surfaces, detailed material analysis was performed on the uncoated and coated fibers with poly(MMA-co-MAA). Several different molar ratios of the monomer were used in the free-radical synthesis reaction to fabricate a range of co-polymeric materials with different chemistries [14]. This strategy provided a mean to exert control over the distribution of surface –COOH groups derived from the MAA segments in the co-polymer compositions. A proper distribution of surface functional groups on the bio-receptive platforms plays a critical role in efficient protein immobilization [22]. Both the lack of sufficient functional groups on the surface or an overly functionalized surface might result in protein deactivation and subsequent unsuccessful biomolecule immobilization [22]. Among the different compositions of poly(MMA-co-MAA) that were used for fiber coating integrated into the clinical assay for enhanced DENV detection, poly(MMA-co-MAA-9:1) has shown a significant performance for virus detection in comparison to other compositions [14]. As the numbers in the bracket suggest, this copolymer composition contains 90% of MMA monomer and only 10% of MAA monomer in its reaction mixture. Assays carried out with this co-polymer composition resulted in the highest signal intensity (six times greater detection signal than conventional assay), along with considerably improved sensitivity, specificity, accuracy, and limit of detection (summarized in Table 1) [14]. 

Our initial work suggested that the 9:1 composition offers an optimal concentration of surface –COOH groups for effective protein immobilization [14]. For that reason, the major focus of the current study was narrowed down to a further analysis of the coated PHB fibers with this specific co-polymer composition in comparison to the pure electrospun PHB fibers.

**Scheme 1:**
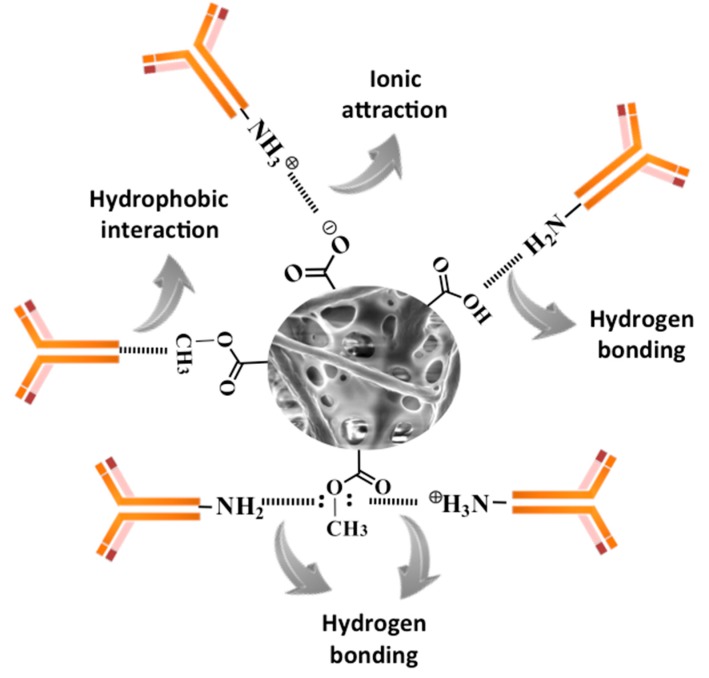
Different physical means through which antibodies can be immobilized on the surface of the chemically designed electrospun PHB fibers.

Scheme 1, in great detail, provides all the possible interactions that might occur between the chemically designed bio-receptive fiber mats and capture antibodies. This scheme clearly emphasizes the importance of the surface –COOH groups introduced through the dip-coating process. Since antibodies are from the family of polypeptides, they often share similar functional groups such as –NH_2_ or –COOH groups. As can be observed from the scheme, surface –COOH groups (in their neutral or amphoteric forms) can involve antibodies in different interaction types such as ionic attraction and/or H-bonding, while tuned wettability of the platform could promote the known force of hydrophobic interaction in attracting biomolecules towards the surface. This scheme presents different means of attaching antibodies to the surface through physical immobilizations, which, in turn, can be crucial for the subsequent detection of DENV.

**Figure 1:**
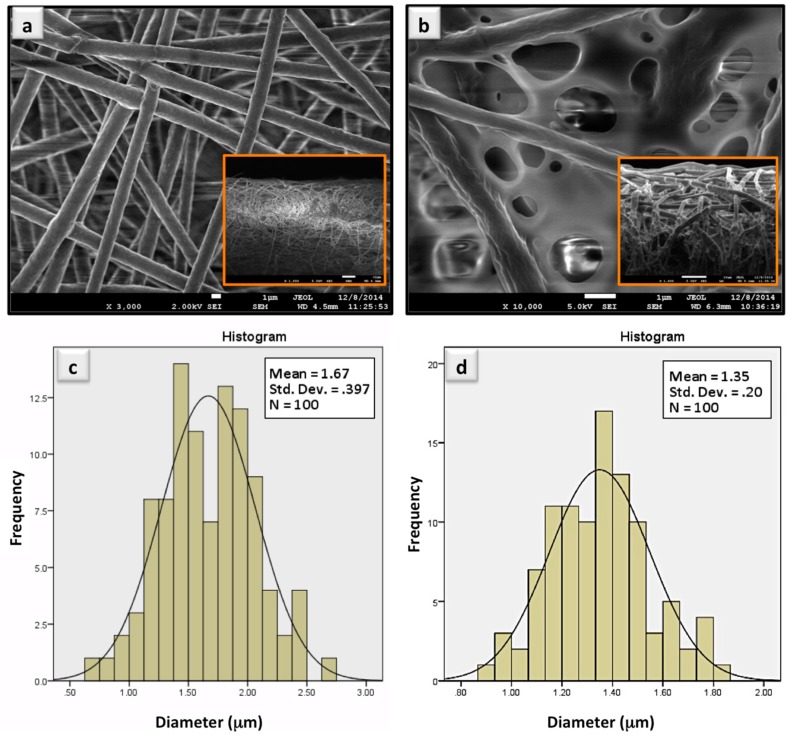
SEM and diameter range analyses of: (**a**,**c**) uncoated; and (**b**,**d**) polymer coated PHB fibers, respectively. Inserts are cross section SEM images of the samples (**a**,**b**).

The morphology of the uncoated and polymer coated electrospun PHB fibers was analyzed by FESEM in both frontal and cross-section views. Typical fiber mat structures can be observed in Figure 1a that display the morphology of PHB fibers. A simple comparison between the frontal views of the pure and coated fibers (Figure 1a,b) and their cross-section views (insets in Figure 1a,b) reveals a distinct difference in the morphology of the analyzed samples. Diameter range analyses of the uncoated (Figure 1c) and coated (Figure 1d) sample reveal a reduction in the average fiber size for the PHB mat after coating in comparison to the uncoated PHB fibers. The average diameter of 1.67 μm for PHB fibers was found to be lower for the coated fibers (1.35 μm), which is most likely due to surface erosion of the fibers with THF as the solvent for the polymer coating. The porosity of the fiber mat, nonetheless, was reduced due to the filling effect of the coating polymer. A partial filling of the interstitial spaces among the network of fibers can also be seen in Figure 1b. The polymer coating reduces the average pore size of the fiber mats from ~12 μm (uncoated fibers) to ~2 μm (coated fibers). While a fiber network offers a considerable surface area for protein interaction, an average reduced pore size does not block such pores, but rather introduces a smaller pore size that may act as a barrier for undesirable phenomenon of protein entrapment [8,14,23,24]. Our previous attempts in optimizing developed polymer coated fiber platforms have shown that uncoated fibers produce a higher false positive signal that, in turn, impacts the performance of the platform when accuracy and specificity is concerned [14,24].

**Figure 2:**
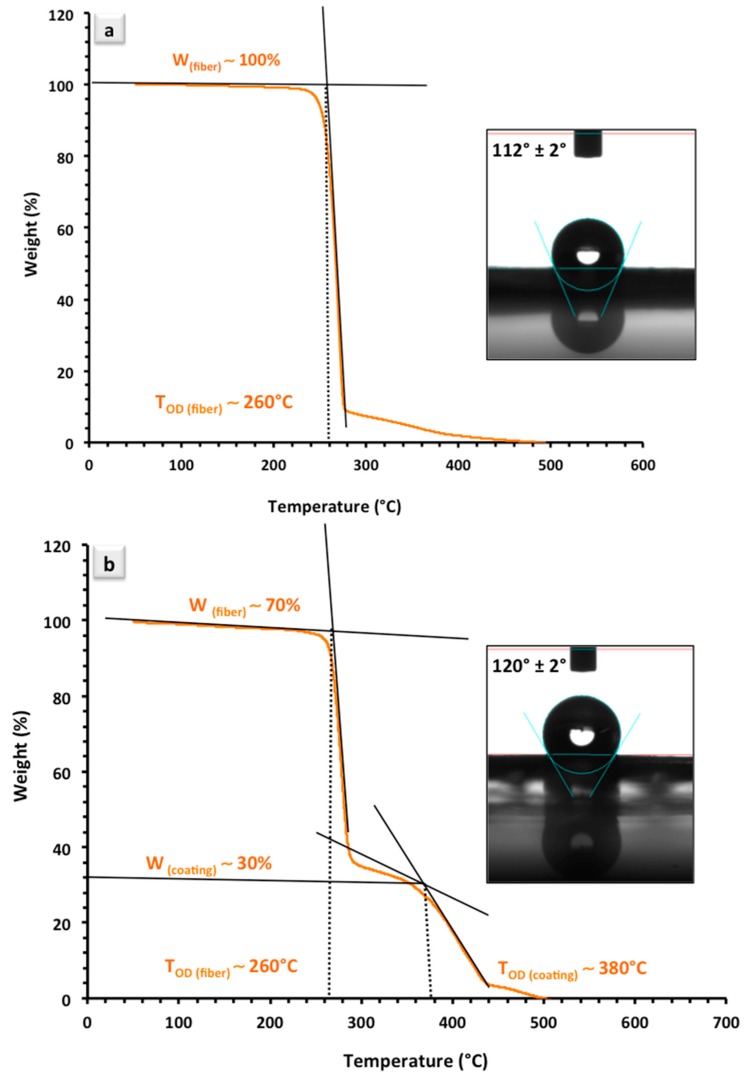
TGA and WCA analyses of: (**a**) uncoated; and (**b**) polymer coated PHB fibers. W and T_OD_ in both TGA plots refer to the weight and temperature for the onset of degradation, respectively.

Apart from the pore size, a higher level of hydrophobicity of the fiber platforms caused by the polymer coating is another advantageous feature. Inserts in Figure 2a,b suggest an increased hydrophobicity of the samples after coating that not only involves antibodies in hydrophobic interaction, but also minimizes the chance of protein entrapment and risk of error in the assay [14]. While co-polymer coated parts of the surface offer –COOH groups, which are known to be hydrophilic in their nature, in contrary, the surface shows an overall improved hydrophobicity. This can be explained through a combined Casie-Baxter/Wenzel state when a large contact angle hysteresis is observed [25]. Under such a circumstance, the formation of Casie–Baxter bridges slows down the transition of water droplets to a fully wetted Wenzel regime [25]. Casie–Baxter bridges are typically formed on chemically heterogeneous surfaces or surfaces containing air pockets [26], which are both present in the case of polymer coated PHB fibers. Due to the presence of an energy barrier, these regimes have separate energy minima. Therefore, the penetration rate for the droplet within the surface matrix is lower.

The chemical composition on the top nano-layers of the PHB, PMMA coated PHB, and poly(MMA-co-MAA) coated PHB fiber mats was analyzed with XPS and the results are presented in Table 2. Observed peak positions for pure PHB fibers are in agreement with the reported data in the literature [27]. Data demonstrated a considerable increase in the peak of C1s after coating, attributed to the presence of PMMA coated segments. The C1s peak assignment at (286 eV) shows a decrease for the coated samples with poly(MMA-co-MAA). This peak assignment is attributed to the carbon atoms of the O–CH_3_ groups known as the main building blocks of MMA monomer units. It is then expected that a lower signal intensity will be recorded for this peak assignment due to the systematic replacement of the MMA with MAA monomer units. Additionally, an obvious increase in the signal intensity of aliphatic carbon (O–C=O, 284 eV) was also detected in the case of poly(MMA-co-MAA) coated PHB fibers compared to PMMA coated PHB fibers. This increase in the number of aliphatic carbon atoms occurs while the O1s peak (530 eV) remains constant for both analyzed samples, which also corresponds to the presence of MAA monomer units, thus providing clear evidence for the existence of –COOH groups on the surface. 

TGA analysis was conducted on the uncoated and coated electrospun PHB fibers for a compositional analysis of the pure and hybrid fiber materials. Figure 2a represents the weight percentage for pure electrospun fibers in which the whole content of the material (~100%) is comprised of PHB. The onset of degradation was found to be at T_OD_ ~260 °C, which is in agreement with previously reported values [28,29]. In Figure 2b, we depict the thermal analysis results of the PHB fibers coated with poly(MMA-co-MAA). Two individual degradation peaks were observed in Figure 2b and the weight percentage of fibers reveals that approximately 70% of the total weight is PHB and ~30% of the overall mass is poly(MMA-co-MAA). Thermal analysis of the uncoated and coated samples along with the observations from the SEM analysis (Figure 1a,b) provide clear confirmation on the successful polymer coating of the electrospun fibers.

As the plot in Figure 2b suggests, the same T_OD_ value was obtained for the onset of degradation as for the PHB fibers. This plot also reveals a second degradation peak at T_OD_ ~380 °C, which is the temperature at which the poly(MMA-co-MAA) coatings began to degrade. A comparison between the thermal degradation of the two compounds indicates that the decomposition of poly(MMA-co-MAA) happens in a higher temperature range than that of PHB. Therefore, it can be concluded that poly(MMA-co-MAA) has improved the thermal stability of the coated fiber platforms, in a general sense [30]. The major weight loss occurs in the range of 300 °C to 440 °C, which corresponds to the structural decomposition of the co-polymer segments. Unlike PHB, the degradation of the co-polymer composition takes place over a broader range of temperature (Figure 2b, second peak). Upon further heating, MAA segments of copolymer can participate in the formation of anhydride groups between pairs of –COOH groups in the decomposing structure of the material [31]. As a result, the degradation peak is flattened by the constant formation and degradation of the anhydride groups in the MAA-containing fibers [32].

**Figure 3:**
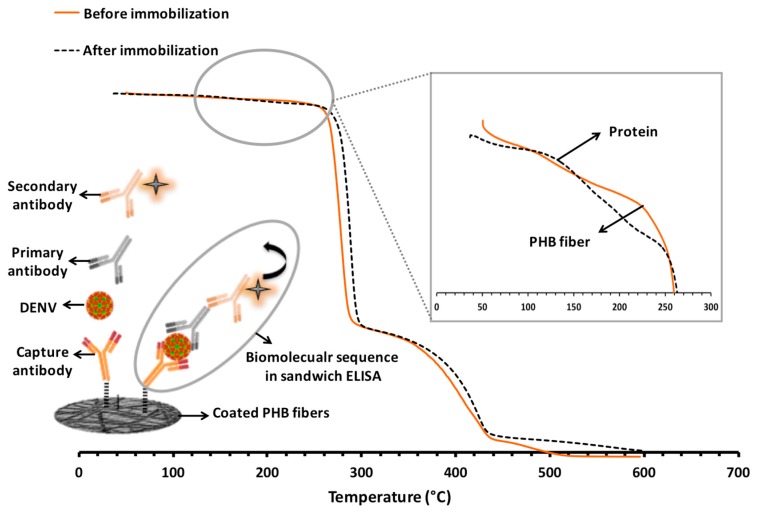
Thermal analyses of the coated PHB fibers before and after protein immobilization, along with a schematic representation of the sandwich ELISA that takes place on the surface of the coated fibers and leads to the final detection signal; inset is the zoomed in region between 0 °C and 300 °C showing the onset of degradation for proteins, as well as PHB fibers.

Electrospun PHB fibers coated with poly(MMA-co-MAA) were also analyzed by TGA immediately after immobilization of the capture antibody against DENV. Figure 3 depicts the thermal analysis of the coated electrospun platform along with a schematic representation of the biomolecular sequence that was immobilized on the surface through a sandwich ELISA [14]. This biomolecular chain that eventually leads to the analytical detection signal, can only take place if the initial step of immobilization has been achieved successfully. Therefore, the presence of capture antibodies on the surface of the coated fibers indirectly corresponds to the quality of DENV detection. The insert in Figure 3 shows the zoomed in area that is marked in the TGA plot. As can be seen in this inset, two separate peaks/shoulders are detected: (i) in the region between 100 °C and 170 °C that is attributed to the degradation of the proteins [33,34,35]; and (ii) in the region between 170 °C and 260 °C, which refers to the decomposition of the fibers (observed in Figure 2a,b). Since both examined samples underwent the same processes (except for the protein immobilization) prior to TGA analysis, the peak/shoulder starting at 100 °C can most likely be assigned to the onset of degradation for the immobilized proteins on the samples. Considering the rate of temperature rise applied in TGA analysis (20 °C/min), it can be concluded that the complete denaturation of the proteins took place within three and a half minutes, which is in line with previously reported data on the thermal degradation of the proteins [33,34,35].

**Figure 4:**
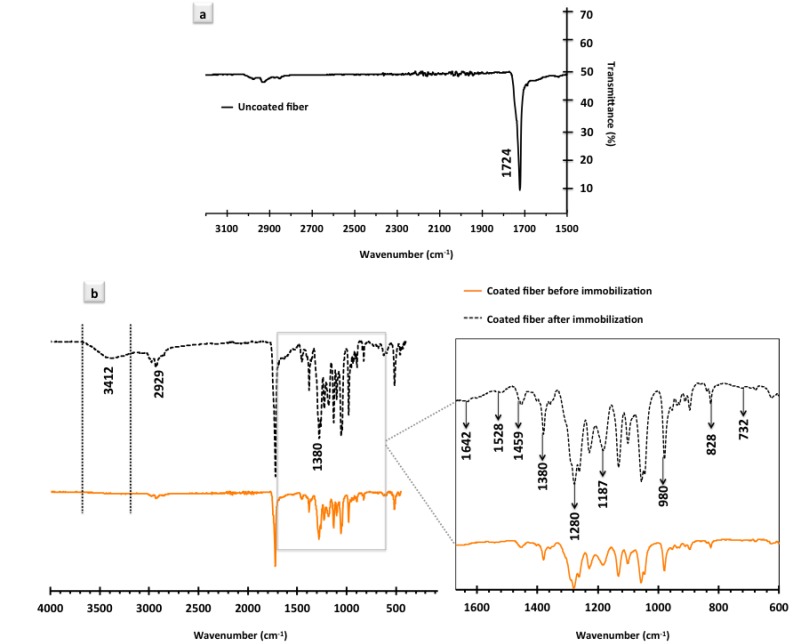
FTIR analysis of the uncoated and coated electrospun PHB fibers: FTIR spectrum of (**a**) pure PHB fibers; (**b**) co-polymer coated PHB fibers before and after protein immobilization. Zoomed-in area is the finger print region of the spectra.

FTIR spectra were recorded for uncoated and coated PHB fibers before and after immobilization of capture antibody against DENV. Figure 4a depicts an FTIR spectrum of pure PHB fibers before coating. This spectrum contains a strong band at 1724 cm^−1^ that is attributed to the C=O stretching of ester/acid carbonyl groups [36]. Absorption bands associated with C=O stretching are usually strong as a large dipole alteration takes place in the vibrational mode [37]. Expectedly, the same sharp peak was also observed in the IR spectrum obtained for coated PHB fibers (Figure 4b). The triple peak at ~2900 cm^−1^ (Figure 4b) corresponds to the C–H stretching vibration within the molecules, while in an overlapping mode, the region at 2800–3100 cm^−1^ can also be attributed to the O–H stretching vibration in –COOH groups derived from MAA segments of the co-polymer [36,38]. The region to the right-hand side of the spectrum (from 400 to 1300 cm^−1^) contains a complicated series of absorptions due to all manners of bending vibrations within the individual molecules. This region is known as the fingerprint region at which it is typically difficult to pick out individual bonds in comparison to the higher wavenumbers regime [39]. Detailed analysis of the fingerprint region (shown in the inset in Figure 4b) reveals peaks that are typical for poly(methyl methacrylate) (PMMA) and poly(methacrylic acid) (PMAA). For instance, the peaks at 1380 cm^−1^ and 1280 cm^−1^ (present in both spectra, before and after protein immobilization) can be attributed to the –CH_2_ bending vibration [40] and C–O stretching, respectively [41]. This particular peak (1280 cm^−1^) is specially known for –CH_2_ and/or –CH_3_ bending vibration in close proximity to C=O [42]. Moreover, the peak at 1459 cm^−1^ can also be assigned to the aliphatic –CH_2_ or –CH_3_ bending vibration [42]. The peaks at 1187 cm^−1^ and 980 cm^−1^ are respectively attributed to the asymmetric stretching of C–O–C and the deformation of C=CH_2_ [43,44], while the peak at 828 cm^−1^ might be assigned to CH_2_ rocking [43].

In the FTIR spectrum of the coated PHB fibers after protein immobilization, there is one obvious peak that does not appear in the spectrum obtained for coated PHB fibers before protein immobilization. This particular peak, which is located at ~3400 cm^−1^, can be attributed to the aliphatic primary amine of antibodies, as it does not exist in the spectrum of the samples, which were not used for protein immobilization. This particular region, in the literature, is also known to correspond to the amide and/or amine N–H stretching vibration of proteins [45]. Additional confirmation for the presence of antibodies was found via the careful analysis of the finger print region. A detailed comparison between the spectra before and after protein immobilization revealed an additional peak/shoulder located at 1642 cm^−1^. In the literature, this region is attributed to both amide I groups of antibodies and the presence of beta sheets in the structure of the proteins [42,46]. Beta sheets are pleated structures inside the biomolecules, which consist of several polypeptide chains in a stretched form that are connected by backbones of hydrogen bonds [47]. A small peak detected at 732 cm^−1^ that again does not exist in the spectrum of the fibers before protein immobilization corresponds to the amid IV groups of the proteins, which is in line with previously reported results [42,48]. An obvious shoulder also appears at 1528 cm^−1^ in the spectrum of the protein immobilized fiber sample that is assigned to the amid II groups of the IgG antibodies. This finding is in agreement with the full spectra of IgG reported in the literature [42,48]. It is important to note that immobilized capture antibodies used for of the virus detection in our experiments were also from the category of IgG antibodies. 

**Figure 5:**
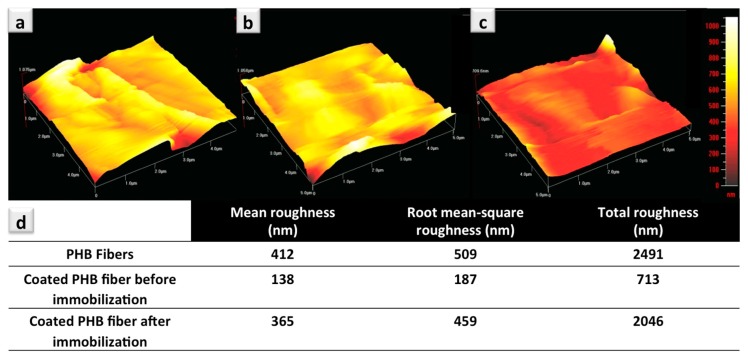
Topography analysis of the uncoated and coated electrospun PHB fibers (dimension of the analyzed area = 5 μm × 5 μm): pure PHB fibers (**a**); co-polymer coated PHB fibers before (**b**) and after protein immobilization (**c**), as well as surface characteristics of the uncoated and polymer coated PHB fibers measured by AFM (**d**).

Surface topography of the uncoated and coated electrospun PHB fibers (before and after protein immobilization) was analyzed by AFM and the results are presented in Figure 5. In Figure 5a, we depict the topography of the pure PHB fibers. A relatively smoother surface was detected for the coated PHB fibers (Figure 5b) in comparison to the pure PHB fibers, while the roughness parameters increased after protein immobilization (Figure 5c). The roughness data are summarized in the Table in part d of Figure 5. From the data in this Table, it is also evident that there is a decrease in the mean, root mean-square, and total roughness of the samples after co-polymer coating [14]. The presence of the proteins immobilized on the coated fibers can also be observed from the topography analysis that considerably alters the surface properties (Figure 5b, after immobilization). After immobilization of the proteins (capture antibodies), all the roughness parameters have been significantly increased (Figure 5d, data in the Table). The same observations have been reported for AFM analysis, confirming the enhancement of the surface roughness resulting from the presence of proteins on the surface of the examined samples [1,49,50,51].

When SEM, AFM, XPS, WCA, TGA, and FTIR analyses are considered together to draw a coherent conclusion for the behavior of the surface, our observations can be summarized as follows:

1. Morphology results obtained from SEM images (Figure 1b) of the coated fiber mats show a considerable coverage between the network of fibers. In AFM analysis, the PMMA coated sample shows a fairly flat topography with considerably reduced surface roughness when compared to the uncoated samples, which is in line with SEM. The same observations were made in our previous publication, indicating decreased surface roughness as a result of coated segments [14,23,24].
2. Another reason for such a decrease in roughness, to a much lower extent, might be due to the presence of surface –COOH functional groups on the co-polymer segments of the coated platforms that do not exist on the surface of the pure PHB fibers. The presence of the –COOH groups imposes an overall softer nature to the coated fiber mat. It is known that –COOH functional groups are relatively hydrophilic in their nature; therefore, they impose the same behavior to the coated fiber platforms [14,19]. Presented XPS data on the Poly(MMA-co-MAA) coated PHB fibers also provide confirmation on the presence of –COOH groups at the topmost layer of the coated surfaces.
3. It is, however, of a great importance to note that the concentration of –COOH groups in this study is not high enough to reduce the WCA of the coated surface, particularly in competition with the Casie–Baxter effect. In our previous study, we examined five different compositions of the same cop-polymer material with varied concentrations of –COOH groups. As the molar ratio of MAA monomers (and subsequently, the concentration of –COOH groups) increased in the samples, both the surface roughness and the contact angle gradually showed major drops, but not with a low concentration of hydrophilic monomer (10%, MAA) [14]. In an extreme case, synthesized material showed a rather gel-like characteristic with a high degree of hydrophilicity as the molar ratio of MAA monomer was increased up to 50% in the reaction mixture [14]. This is in line with the commonly acknowledged phenomenon that in hydrophilic materials the surface roughness decreases with the contact angle [15,52]. Nonetheless, in the WCA study of (9:1) co-polymer composition presented here, the effect of hydrophilic –COOH groups is negligible and the increased WCA for coated fiber mats can be mainly explained by the coverage of the interstitial spaces among the matrix of fibers.
4. AFM analysis, hand in hand with TGA and FTIR analyses, tracks down the presence of antibodies on the surface of the fiber-based bio-sensing platform in an effective manner. Changes in the surface topography recorded by AFM, an additional shoulder on the TGA spectrum attributed to the presence of antibodies, and the appearance of several different picks in the FTIR spectrum are clear confirmation of the presence of antibodies on the coated fiber mats.

Confirmation analyses on successful immobilization of the biomolecular entities play a pivotal role in the development of an efficient bio-receptive platform for biosensor applications. Further attempts will be directed to the detailed analysis and quantification of the protein attachment/conjugation on the surface of the coated fibers and possibly protein entrapment within the matrix of fibers. 

## 4. Conclusions

Co-polymer coated electrospun PHB fibers were used for the immobilization of dengue capture antibodies. The developed platforms have been thoroughly examined before and after the initial step of protein immobilization by different characterization techniques such SEM, TGA, WCA, FTIR, and AFM to obtain confirmation not only of the successful co-polymer coating of the fibers, but also of the presence of protein on the developed surfaces. Clear signs for the existence of antibodies on the network of coated PHB fibers were recorded in a thermal analysis of the samples. FTIR analysis has provided detailed confirmation of the presence of the proteins on the surface, especially in the finger print region. Moreover, a clear topographical alteration and significantly increased surface roughness of the bio-receptive platforms confirmed the existence of antibodies on the surface. Apart from the measurement of the detection signal intensity, which results in an indirect validation of protein immobilization, the application of the aforementioned techniques provides valuable information regarding the biomolecular presence on the developed bio-receptive surfaces. 

## Figures and Tables

**Table 1:** Sensitivity, specificity, accuracy, and limit of detection (LoD) for coated and uncoated electrospun PHB fibers.

Type of Fibers	Sensitivity (%)	Specificity (%)	Accuracy (%)	Limit of Detection (LoD, p.f.u × 10^3^/mL)
PHB	93	30	70	2.163
PMMA coated PHB	100	60	85	0.541
Poly(MMA-co-MAA) coated PHB	100	80	88	0.008

**Table 2:** Surface concentration (%) investigation by XPS on coated and uncoated electrospun PHB fibers.

Peak		C1s		O1s
Peak assignment	–CH	–CO	O–C=O	
Binding energy (eV)	284	286	288	530
PHB (%)	22	14	3	20
PMMA coated PHB (%)	42	16	12	26
Poly(MMA-co-MAA) coated PHB (%)	44	13	15	26
